# Serology of Viral Infections and Tuberculosis Screening in an IBD Population Referred to a Tertiary Centre of Southern Italy

**DOI:** 10.1155/2017/4139656

**Published:** 2017-09-17

**Authors:** Marco Ardesia, Giuseppe Costantino, Placido Mondello, Angela Alibrandi, Walter Fries

**Affiliations:** ^1^Department of Clinical and Experimental Medicine, Clinical Unit for Chronic Bowel Disorders, IBD-Unit, University of Messina, Messina, Italy; ^2^Infectious Diseases, Department of Human Pathology, University Hospital of Messina, Messina, Italy; ^3^Department of Economics, University of Messina, Messina, Italy

## Abstract

**Background:**

With the introduction of more potent immunosuppressive agents in inflammatory bowel disease, prevention of opportunistic infections has become necessary by introducing screening programs. Prevalence of the most important infectious agents may vary in different geographical areas. The aim of our study was to assess the immune status for hepatitis B, varicella, mononucleosis, and cytomegalovirus infection together with the determination of the hepatitis C and tuberculosis status in Southern Italy.

**Methods:**

Prevalence of latent tuberculosis, together with serology of hepatitis B and C, Epstein-Barr virus, varicella zoster, and cytomegalovirus were collected by analysing retrospectively the clinical charts of IBD patients. Data were integrated with demographic and clinical features.

**Results:**

Data from 509 IBD patients divided in two age groups showed a prevalence of HBV infection in nonvaccinated patients of 9%. Seroprotection (HBsAb) in vaccinated IBD patients was lower (*p* < 0.0001) compared with that in controls. Prevalences of herpesvirus infections fluctuate between 51% (CMV) and 85% (EBV) and 84% (VZV) in younger patients. Latent tuberculosis and hepatitis C infection were found only in patients > 37 years of age.

**Conclusions:**

In younger patients, high susceptibility rates for primary herpesvirus infections should determine the choice of treatment. Loss of HBV seroprotection in already vaccinated patients should be considered for booster vaccination programs.

## 1. Introduction

Inflammatory bowel diseases (IBD) are diseases of yet unknown etiology characterized by chronic inflammation of the gastrointestinal tract resulting from an interaction between the immune system and environmental risk factors [1, 2]. Medical therapy is based mainly on drugs aiming to reduce the inflammatory response by acting on the immune system, such as corticosteroids, immunomodulators, and biological drugs [3–6]. The immune system performs its activities not only versus infectious agents but also on tumour surveillance [7]; thus, a reduction of the immune system activity by immunosuppressive drug may expose the patient to an increased risk of infection and tumor growth. According to the choice of immunosuppressive therapy to treat IBD, the risk of infections increases not only globally especially when used in association [8] but specific treatments may expose the patients to specific risks, for example, tuberculosis reactivation with anti-TNF agents, bacterial infections with anti-TNFs and steroids, or viral infections with thiopurine use [9]. Not only primary infections with a more severe clinical course in immunosuppressed patients have been reported, especially with varicella zoster virus (VZV), cytomegalovirus (CMV) [10], and Epstein-Barr virus (EBV), the latter can also promote lymphoproliferative disorders [11, 12], but also reactivation of latent infections should be considered such as tuberculosis (TBC), hepatitis B (HBV) and C (HCV), VZV, CMV, and EBV.

Based on these considerations, the European Crohn's Colitis Organization (ECCO) has recently published the guidelines for the management of opportunistic infections in patients with IBD [13] in order to reduce the risk of infections, reactivation of latent infections, and the possible onset of neoplastic disorders mediated by infections.

Since the prevalence of infections is variable according to the different geographic regions, the knowledge of the epidemiology in a specific geographic area may play an important role to address screening programs and management of IBD. The aim of the present study was to assess the prevalence of viral and mycobacterial infections in a population of patients referred to an IBD tertiary centre.

## 2. Material and Methods

### 2.1. Patient Population

The IBD unit at the University Hospital of Messina is the only IBD referral centre in the province of Messina which covers an area with a population of roughly 600,000 people. However, patients from other Sicilian provinces from the East Coast and from the South Calabria are followed by our centre for first diagnosis and/or for follow-up. The study was approved by the Ethics Committee of the University Hospital of Messina (protocol 50/17). Data were collected and handled anonymously according to the national law on data protection.

We retrospectively revised the outpatient charts from 2009 to 2016. Patients were routinely analysed at their first visit at our centre (i.e., at their first diagnosis in our clinic or patients already diagnosed and followed at other hospitals or general practitioners). Anamnestic data on the vaccination status for hepatitis B (HBV) and for varicella (VZV) or previous infections by VZV, HBV, or HCV were recorded. Serologic data concerning active or previous infections due to HBV (HBsAg, HBsAb, and HBcAb), HCV (HCVAb), EBV (VCA-IgG), VZV (VZV-IgG), CMV (CMV-IgG), and TBC (Mantoux test and/or QuantiFERON-TB Gold test and/or chest X-ray) (Mantoux test: tuberculin units of purified protein derivative, Tubertest, Sano Pasteur, MSD, Lyon, France; QuantiFERON-TB Gold test: QuantiFERON-TB Gold, Qiagen, Hilden, Germany) were collected.

Contemporarily, we also registered the following data: gender, age, disease (ulcerative colitis, Crohn's disease, and IBD undefined (IBDU)), Montreal classification [14] prior to surgery (for patients with CD), year of diagnosis, time elapsed between diagnosis and infectious diseases screening, and therapies prior to screening. Furthermore, in consideration of the literature data concerning a more frequent loss of HBV vaccine titre in patients with IBD compared to the general population [15–17], the serologic status for HBV (HBsAb) in patients aged ≤37 years in 2016 (patients born later than 1979), that is, a population subjected to mandatory vaccination for hepatitis B in Italy (for vaccination strategy in Italy, see Zanetti et al. [18]), was compared with that of a healthy control population (without IBD, no previous HBV infection, age ≤ 37 years) by retrospectively revising the charts of hospital employees subjected to regular working ability visits at the Occupational Medicine Unit of the University Hospital of Messina.

### 2.2. Definitions of Present or Past Infection

Patients who tested HBsAb positive but negative for HBcAb and HBsAg were considered vaccinated (confirmed by interview). Patients aged ≤37 years and negative for HBsAg, HBcAb, and HBsAb ≤ 10 mIU/mL were considered vaccinated and who have lost the protective vaccination titre (in this case, HBsAb was considered negative), while an HBsAb titre > 10 mIU/mL was recognized as positive. HBcAb positivity was followed by HBV-DNA testing in order to exclude active replication. Patients positive for VCA-IgG EBV, VZV-IgG, and CMV-IgG were considered as patients who have had a previous viral contact and thus positive for statistical analysis. HCVAb positivity was followed by HCV-RNA testing in order to exclude active replication. Either QuantiFERON-TB Gold or Mantoux test positivity, in the absence of active lesions identified at chest X-ray, fever, and cough, was interpreted as latent TBC (positive value at statistical analysis).

### 2.3. Subgroups for Statistical Analysis

As for HBV infections, we arbitrarily divided patients into 2 age groups, that is, age ≤ 37 years and >37 years, accordingly, for all variables for the statistical analysis. Moreover, for CMV and EBV infections, we expressed the positivity for previous infections in 5-year intervals in order to evaluate rates of seroconversion in the various age classes.

### 2.4. Statistical Analysis

Categorical variables were expressed as absolute frequency and percentage and numeric variables as mean ± standard deviation or median (minimum and maximum). The Kolmogorov-Smirnov test was carried out to test for normal distribution of the numeric variables (age (expressed in years) and duration of disease (expressed in months)). In order to compare UC and CD patients, chi-square or Fisher's exact test was employed for categorical variables. For numeric variables, was used the Mann–Whitney test. The associations between categorical variables were evaluated by chi-square test, or Fisher's test when appropriate. An existing interdependency between age, duration of disease, and all other investigated variables was verified using the nonparametric Spearman's test. Potential predictive factors such as gender, age, type of disease, therapy, disease duration for a positive or negative testing result, and univariate logistic regression models were used. On the basis of the obtained results, multivariate models have been estimated for the same outcome, inserting as covariates all statistically significant variables in the univariate models, in order to identify significant independent predictors by outcome analyzed. Statistical analysis was performed using the statistical software SPSS for Windows, version 17.0. A *p* value < 0.05 was considered statistically significant.

## 3. Results

The charts from 1293 IBD patients were reviewed. Data from 509 patients screened for biologic and/or thiopurine therapy were analysed. Demographic and clinical characteristics of the study cohort are reported in Table 1. Two patients with IBDU were included in the UC group. No patient reported vaccination for the TBC. Roughly 80% of patients reported clinical varicella, but vaccination for varicella was reported in only 9 patients. Disease activity at the moment of screening was variable ranging from mild to moderate/severe disease, and former steroid treatment at any time point was reported by 86% of all patients, immunomodulators by 34%, and biological therapies (anti-TNF*α*) by 15%.

Data about immunization status of all viral serological markers and TBC screening are reported in Tables 2 and 3. Concerning data of HBV and HCV viral infections, information were available from 82% of the samples (HBsAb: 87%, HBsAg: 88%, HBcAb: 82%, and HCVAb: 86%), while for the herpesvirus EBV, VZV, and CMV, data were available from 66%, 50%, and 64% of all patients, respectively. Tuberculosis screening data, Mantoux, and/or QuantiFERON-TB Gold were available from 315 patients (62%).

### 3.1. HBV and HCV Infection

Concerning HBV, in our nonvaccinated population, that is, patients aged >37 years, HBV infection was found in 9.6% and 8.4% in CD and UC, respectively (Table 2). In this age group, HBsAg carriers were 2% and 1.6% in CD and UC, respectively. In the patient group aged ≤37 years, 5 patients (3 CD, 2 UC) were asymptomatic carriers or infected by HBV (HBV-DNA negative in all 5 patients). There was no statistical difference in the prevalence for HBV infection between CD and UC or between different Montreal phenotypes. In CD patients, there was no association between former surgery and HBV infections.

Dividing the sample into 2 age groups according to the mandatory HBV vaccination (age ≤ 37 years versus age > 37 years), we found a significant difference for HBcAb (*p* = 0.008), while no difference was found for HBsAg prevalence (*p* = 0.631). At univariate logistic regression, there was a significant association between HBsAb and HBcAb positivity and age (*p* < 0.0001, for both). No association was found for gender and former treatments (steroids, immunomodulators, and biologics).

**Figure 1:**
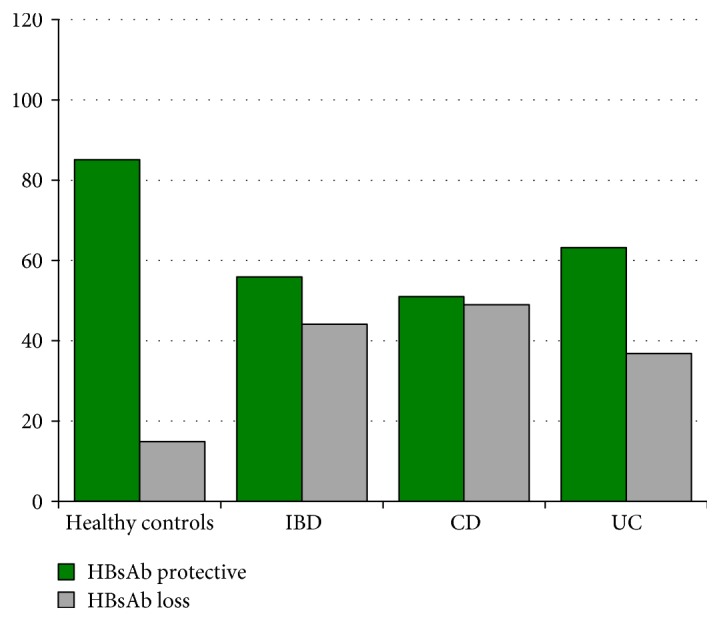
Comparison of HBsAb positivity between IBD patients aged ≤37 years and healthy controls aged ≤37 years. HbsAb positivity: healthy controls versus IBD *p* < 0.0001.

Moreover, to evaluate the persistence of HBsAb in vaccinated IBD patients, we carried out a comparison with 174 healthy controls aged ≤37 years (median age: 31 years (26–37 years); males: 69) (Figure 1) excluding the 5 IBD patients with confirmed HBV infection from the statistical analysis. In subjects aged less than 37 years, there was a significant difference in HBsAb positivity between IBD and controls with a positivity of HBsAb of 55.9% in IBD patients and of 85.1% in controls (*p* < 0.0001). Although numerically with lower values, statistically CD patients behaved no different from UC (HBsAb +ve: 51% versus 63.2%).

HCV infection (HCVAb positivity) was found in 2.5% of all patients (CD: 1.2%, UC: 4.2%) but was virtually absent in IBD patients aged ≤37 years as well as in controls (data not shown). Considering patients aged >37 years, the overall HCVAb positivity rose to 4.1% (Table 2). There was no statistical difference between CD and UC (*p* = 0.065; OR 3.541; 95% CI 0.93–13.53) and no association between HCVAb positivity and Montreal phenotype in CD and UC, or surgery in CD. There was a statistical difference for HCVAb between the groups ≤37 years and >37 years (*p* = 0.008). At univariate logistic regression, we found an association between HCVAb and age (*p* < 0.0001; OR 1.11; 95% CI 1.05–1.17) and former steroid use (*p* = 0.005; OR 0.174; 95% CI 0.05–0.59) but not with gender, former nonsteroidal therapies, and surgery (in CD). All results were confirmed by multivariate logistic regression (age: *p* < 0.0001, OR 1.11, 95% CI 1.05–1.17; steroid use: *p* = 0.006, OR 0.15, 95% CI 0.04–0.59).

### 3.2. Herpesvirus Infection

Past infection by EBV was found in 90.5% in the whole cohort (CD: 88.4%, UC: 93.2%) (Table 3). There was no difference between CD and UC. There was a statistical difference between the 2 age groups, ≤ 37 years and >37 years (*p* = 0.006). At univariate logistic regression, we found an association with age (*p* = 0.006, OR 1.038, 95% CI 1.011–1.066) but no association with Montreal phenotype, former therapies, surgery in CD, and gender (data not shown). Considering EBV seropositivity by age intervals (Supplementary Figure 1 available online at https://doi.org/10.1155/2017/4139656), a 100% positivity was reached only by patients over 60 years old (Supplementary Figure 1; in this figure, reference values from [19] were included).

Concerning CMV, CMVAb positivity was found in 66.5% in the whole IBD cohort (CD: 63.7%, UC: 69.9%) (Table 3). There was no difference between CD and UC. At univariate logistic regression, we found statistical association with age (*p* < 0.0001, OR 1.048, 95% CI 1.031–1.066), gender (*p* = 0.034, OR 1.69, 95% CI 1.04–2.73), with greater positivity of serological markers in women, and longer disease duration (*p* = 0.015, OR 1.003, 95% CI 1.001–1.006); however, at multivariate logistic regression, significance was confirmed only for age (*p* < 0.001, OR 1.047, 95% CI 1.028–1.066) and female gender (*p* = 0.022, OR 1.81, 95% CI 1.09–3.01). No association was found with Montreal phenotype, former therapies, and surgery in CD. In Supplementary Figure 2, CMV seropositivity is given by age intervals.

Finally, VZV past infection (VZVAb positivity) was found in 90.2% of the whole cohort (CD: 88.9%, UC: 92%). There was no difference between CD and UC and no association with the Montreal phenotype. There was a statistical difference between ≤37 years and >37 years age groups (*p* = 0.003). At univariate logistic regression, we found an association with age (*p* = 0.005, OR 1.047, 95% CI 1.014–1.080) and steroid use (*p* = 0.041, OR 2.71, 95% CI 1.04–7.04), but not with gender, former immunosuppressive therapies, or surgery. At multivariate logistic regression, only age preserved statistical significance (*p* = 0.006, OR 1.047, 95% CI 1.014–1.080). There was a strong concordance (*p* < 0.0001) between serological (VZVAb) and anamnestic data, the latter obtained during follow-up visits.

### 3.3. Tuberculosis

Latent or active tuberculosis was evaluated by means of the Mantoux skin test (233 patients) and/or QuantiFERON-TB Gold (97 patients) (in 15 patients both, the Mantoux skin test and QuantiFERON-TB Gold were carried out). Latent TBC infection was found in 6.7% (21/315) of the whole IBD cohort (CD: 7.8%, UC: 4.9%), but it was virtually absent in subjects <37 years. Two QuantiFERON-TB Gold tests (1 CD, 1 UC) resulted indeterminate. There was no difference between CD and UC and no association with the Montreal phenotype. There was a statistical significance between ≤37 years and >37 years aged group (all TBC evaluation methods: *p* < 0.0001). At univariate logistic regression, we found an association with age (*p* < 0.001, OR 1.06, 95% CI 1.029–1.092) but not with gender, former therapies, and surgery (in CD). Evaluating the concordance rate between Mantoux test and QuantiFERON-TB Gold in 15 patients in which both data were available (8 CD, 7 UC), a concordance of 86.7% (13/15) was achieved.

## 4. Discussion

To the best of our knowledge, this is the first study in Italy that addresses the immune status against different opportunistic infections within a large single cohort of IBD patients. Moreover, we specifically investigated HBV seroprotection with age-matched controls.

### 4.1. Hepatitis Virus

In the present study, we found a similar prevalence of past HBV infection (HBcAb positivity) in our IBD patients compared to that of the general population investigated in a small town in South Italy (HBcAb positivity: 9.0% versus 11.2%) [20], while a nationwide multicentre study conducted in 2001 in Italy showed a prevalence of 10.9% in CD and 11.5% in UC, significantly higher than that in the healthy control population (5.1%) [21]. Finally, latest Italian data revealed lower HBV infection rates in IBD patients (HBcAb +ve 7.3%), similar to that of the general population [22]. In France, the prevalence of HBV infection rates was similar in IBD patients compared with the general population (CD: HBcAb +ve 2.78%; UC: HBcAb +ve 1.59%) [23], while in Spain, the prevalence of HBV infection was lower in patients with IBD compared to the general population [24]. Conversely, a study conducted in China showed past and present infection rates of roughly 40% in IBD patients compared with rates of 27.6% in non-IBD subjects [25].

From our data, it appears that the risk of HBV infection increases with age; however, this result is surely influenced by the mandatory HBV vaccination. Another important issue is the relationship between IBD and HBsAb status in vaccinated IBD patients; we confirm the finding of Waszczuk et al. [17] who reported a seroprotection rate of 54%, underlining that our patients were vaccinated long before the diagnosis of IBD was made. This confirms our previous finding in a much smaller cohort [26].

Among yet identified risk factors for a missing seroprotection despite vaccination are diagnosis if CD, disease duration of more than 7 years, and an age at diagnosis of over 31 years [23], while conflicting data exist about the influence of immunosuppressive drugs on response to and on persistence of vaccine efficacy [15, 27, 28].

Data from this study comparing the IBD population with a healthy population of the same age and geographical area confirm the significant loss of HBsAb titre among patients with IBD, with a greater tendency in CD than in UC (although the statistical significance was not achieved, most probably due to the sample size), as already reported in other studies [27, 28].

Among our vaccinated patients, 5/176 (2.8%) were infected by HBV, a finding consistent with data from Poland [17]. Possible explanation may include an HBsAb titre loss due to immunosuppressive therapies, a not perfectly performed vaccination or a not fully responsive immune system.

HCV prevalence rates in our cohort showed 2.5% in the whole series; this figure rose to 4.1% when we considered patients > 37 years of age. Our data are similar to those of French and Spanish with modest variations [23, 24]. A low prevalence was reported in Chinese patients (0.42%) [25]. In an Italian study from 2001, an HCVAb positivity rate of 7.4% was reported in CD and 0.6% in UC [21], while in our study, we reported a lower positivity rate in CD but a greater proportion in UC but without statistical significance (2.5% all IBD, 1.2% in CD, 4.2% in UC).

Interestingly, in our patients below 37 years of age, no positivity was found indicating an important reduction of HCV infection at least in patients not belonging to specific high-risk groups, like i.v. drug abusers and dialysis patients. This reduction is evident if we consider reference data on the whole population in a Sicilian township obtained 15 years ago reporting a prevalence of 10.4% [20].

### 4.2. Herpesvirus

Epstein-Barr virus (EBV) is a member of the herpesvirus family, a worldwide virus with a prevalence of over 90% in the adult population [29]. After infection, EBV remains in immune cells and is susceptible to reactivation. Few studies are available regarding the epidemiological situation of this infection in IBD. In the present study, patients below 37 years of age presented with a measurable antibody titre of 82.9–88.5%; thus, a primary EBV infection may be possible in up to 20% of all patients in this age group. In a Canadian cohort of 263, IBD patients, seropositivity for EBV reached 100% much earlier starting from age 25 years, [19] without differences between UC and CD. In a Spanish IBD population [30] with a median age of 44 years, EBV seropositivity was found in 96.4%, consistent with our data. In a follow-up study, also Spanish IBD patients reached 20 years earlier than our patients' 100% seropositivity [31]. These latter authors followed seroconversion of nonprotected patients under different treatment regimens. In follow-up, 31% presented seroconversion and, in patients aged >35 years, 1 case of the potentially fatal hemophagocytic lymphohistiocytosis (HLH or macrophage activation syndrome (MAS)) and 1 case of a fatal large B-cell lymphoma were observed, both conditions associated with a primary EBV infection while under immunosuppressive therapy [31]. Primary EBV infection in immunosuppressed IBD patients is involved in lymphoproliferative disorders and HLH, especially in patients treated with thiopurines. Indeed, in a very recent prospective pediatric study, 4 cases of HLH triggered by primary EBV infection were reported and all cases were associated with thiopurine treatment [32]. However, other cases of HLH were also reported with reactivation of latent EBV infection [9]. For these reasons, in the current ECCO guidelines on opportunistic infections [13], screening for EBV should be considered before the start of immunomodulator therapy. Our cohort included patients screened before these last edition of guidelines (in the first edition [33], “screening for latent or subclinical EBV infection … was not recommended”) and among 32/336 EBV negative patients, 10 patients (31%) did receive treatment with immunomodulators with a potential risk for HLH or malignancy.

CMV is a ubiquitous herpesvirus whose prevalence rate in the population varies between 30% and 90%, increasing with age; primary infection is usually asymptomatic in the immunocompetent host and subsequently to it, the virus establishes a latent infection with periodic reactivations [34]. In our patients, CMVAb positivity never reached more than 90%, with a rough mean positivity of 65% until age 50 years which is consistent with Spanish data [30]. Compared to European data, in Central China, a higher prevalence rate for CMV-DNA (84%) and CMVAb-IgG (76%) have been reported in IBD patients aged 40 years, with significantly lower rates in the general population (59.7% and 50.7%, resp.) [35]. CMV superinfection in patients with UC is a well-known problem leading frequently to refractoriness of disease and worse outcomes [36, 37], but why CMV is more frequent in UC than in CD is still matter of debate [38]. In our patients, UC and CD presented with similar rates of positivity in both age groups above and below age 37 years. Similar to EBV, not only primary CMV infections but also reactivations may lead to HLH as formerly reported, especially under thiopurine therapy [9, 32], so screening for unexposed patients may be useful in order to choose safer therapies in not protected patients. This issue is still unaddressed in the ECCO guidelines where screening for CMV is still deemed unnecessary [13]. The relationship between primary CMV infections and HLH or pneumonia [10] is still to substantiate, but considering that below age 37 years less than 60% show seropositivity, it is likely. In our cohort, among CMV seronegative patients, almost 71% (78/110) received immunomodulators; therefore, they may be considered potentially at risk for primary CMV infection-related complications. In follow-up, 1 male CMV-negative UC patient under thiopurine therapy developed fever without worsening of intestinal disease; a primary CMV infection was diagnosed, and the patient recovered uneventful under treatment with ganciclovir, whereas in a former report, a probable primary CMV infection has led to HLH in a 32-year-old female with UC treated with thiopurines [39].

Finally, VZV is an exclusively neurotropic human herpesvirus which causes a primary infection in the skin known as chickenpox. Following primary infection, the virus is located in the basal ganglia of the cranial, dorsal, or autonomic nerve and, as a result of reactivation, it gives rise to a new cutaneous manifestation characterized by rash and pain known as H. zoster or shingles [40]. Our adult patients have an overall VZVAb positivity of 90% (92% in UC), whereas a study conducted on children and adolescents with IBD in the US showed an immunity of only 77% to VZV, emphasizing the need for serological screening in patients with IBD at diagnosis [41], since primary infection with VZV is associated with more severe disease and fatal cases, especially in immunosuppressed patients [42].

We can confirm in our patients that a positive clinical history for chickenpox is strongly concordant with detectable VZVAb, so screening may be limited to those with negative or uncertain anamnestic information in line with the most recent ECCO guidelines concerning VZV [13].

### 4.3. Tuberculosis

Our patients presented with a mean prevalence of latent TBC in 11% of patients aged >37 years, whereas below this age, no positivity was found. Our findings are significantly lower compared with those of another Italian study in which IBD patients with a mean age of 40 years had almost 20%, yielded positive results [43]. A possible explanation for this difference may be that the studied population of Andrisani et al. was recruited in Rome, a city with more tourism and with a higher migration flow. It has also been observed in another European capital, that is, Berlin, that immigration represents a risk factor for TBC spread [44]. In Turkey, another mediterranean country, a retrospective study in an anti-TNF-treated IBD population showed a prevalence of latent tuberculosis of 59% and, during the follow-up period, a prevalence of 5% of active tuberculosis [45]. TBC is an important issue in IBD as shown by a retrospective study conducted on the British general practitioners' databases reporting an annual incidence of active tuberculosis of 20/100,000 in IBD patients compared to 9/100,000 in the control group. A relevant risk of active tuberculosis in patients with IBD was attributed to immunosuppressive drugs and to immunomodulators [46]. Current guidelines state that screening for tuberculosis should be performed by history, chest X-ray, tuberculin skin test, and QuantiFERON tests at diagnosis and before starting therapy with anti-TNF*α* [13]. QuantiFERON-TB Gold test is the preferred test in vaccinated patients, but vaccination against TBC is optional and recommended only for certain risk groups.

## 5. Conclusion

In conclusion, screening should be performed at diagnosis in order to proceed, in case of missing protection, with appropriate vaccination or booster administrations, where available. A potential limitation of the study is that the assessments have been performed, in part, in patients who had already undergone former immunomodulatory and/or anti-TNF*α* therapy. Although no necessary steroid or immunosuppressive treatment was performed during screening, this factor could have affected the outcome of the immune status.

In our patients aged below 37 years, susceptibility for primary infections by EBV and CMV reached 20 to 50%, respectively. A lack of seroprotection should guide the clinician's choice of the most appropriate but potentially less harmful therapy. VZV susceptibility in this younger age group reached also 20% but may be overcome by vaccination; however, screening may be limited to those with negative medical history for chickenpox or shingles. Concerning HBV, there is a significantly lower immune response to vaccination in patients who subsequently develop IBD, numerically more important in future CD patients, although we cannot exclude a loss of seroprotection by immunosuppressive therapies. HCV positivity, although a minor clinical problem, is virtually absent in younger IBD patients. Finally, latent TBC, despite its absence in younger patients, remains an important issue in patients aged >37 years.

## Supplementary Material

Supplementary Fig. 1: Seroprevalence of EBV infection by age intervals; green bars are data from our centre, grey bars reference values from Linton et al [19]. Supplementary Fig. 2: Seroprevalence of CMV infection by age intervals.

## Figures and Tables

**Table 1:** Demographic and clinical characteristics. Data are crude numbers (percentages) or median (min-max). UC: ulcerative colitis; CD: Crohn's disease; 5-ASA: mesalazine; steroids (topical and/or systemic); IMM: immunomodulators (azathioprine, 6-mercaptoruine, and methotrexate); biologics: anti-TNF*α* (infliximab and adalimumab).

Number of patients included	509
Gender, M/F	300/209
Age, median (range), years	42 (17–87)
Disease, CD/UC	289 (56.8%)/220 (43.2%)
(i) CD: M/F	179/110
(ii) CD: age, median (range), years	42 (17–81)
(iii) CD (Montreal classification)	
L1	152 (52.6%)
L2	39 (13.5%)
L3	98 (33.9%)
L4	8 (2.8%)
(i) UC: M/F	121/99
(ii) UC: age, median (range), years	42 (17–87)
(iii) UC (Montreal classification)	
E1	19 (8.6%)
E2	111 (50.5%)
E3	90 (40.9%)
Duration of disease, median (range), months	
(i) Total population	56 (0–612)
(ii) CD	60 (0–612)
(iii) UC	48 (0–600)
Drug treatment (before screening)	
(i) 5-ASA (%) (total/CD/UC)	465 (91.4%)/258 (89.3%)/207 (94.1%)
(ii) Steroids (%) (total/CD/UC)	439 (86.2%)/244 (84.4%)/195 (88.6%)
(iii) IMM (%) (total/CD/UC)	174 (34.2%)/106 (36.7%)/68 (30.9%)
(iv) Biologics (%) (total/CD/UC)	80 (15.7%)/54 (18.7)/26 (11.8%)

**Table 2:** Comparison between patients aged ≤37 years (group 1) and patients aged >37 years (group 2). For all variables—positive values. HBV: hepatitis B virus; HCV: hepatitis C virus; HBsAb: antibodies antisurface antigen HBV; HBsAg: surface antigen HBV; HBcAb: antibodies anticore HBV. ^∗^Statistical significance influenced by vaccination in group 1.

	Group 1 (≤37 years; vaccinated for HBV)			Group 2 (>37 years)			*p* value (total, group 1 versus group 2)
	Total (% +ve)	CD (% +ve)	UC (% +ve)	Total (% +ve)	CD (% +ve)	UC (% +ve)	
HBsAb	99/176 (56.3%)	54/105 (51.4%)	45/71 (63.4%)	35/233 (13.1%)	20/146 (13.7%)	15/122 (12.3%)	*p* < 0.0001^∗^
HBsAg	3/173 (1.7%)	2/101 (2%)	1/72 (1.4%)	5/277 (1.8%)	3/150 (2%)	2/127 (1.6%)	*p* = 0.631
HBcAb	4/161 (2.5%)	2/92 (2.2%)	2/69 (2.9%)	23/255 (9.0%)	13/136 (9.6%)	10/119 (8.4%)	*p* = 0.008
HCVAb	0/169 (0%)	0/101 (0%)	0/68 (0%)	11/268 (4.1%)	3/145 (2.1%)	8/123 (6.5%)	*p* = 0.008

**Table 3:** Comparison between patients aged ≤37 years (group 1) and patients aged >37 years (group 2). For all variables—positive values. EBV: Epstein-Barr virus; VZV: varicella zoster virus; CMV: cytomegalovirus; TBC: tuberculosis (Mantoux skin test or QuantiFERON-TB Gold).

	Group 1 (≤37 years)			Group 2 (>37 years)			*p* value (total, group 1 versus group 2)
	Total (% +ve)	CD (% +ve)	UC (% +ve)	Total (% +ve)	CD (% +ve)	UC (% +ve)	
VCA-IgG EBV	114/134 (85.1%)	68/82 (82.9%)	46/52 (88.5%)	190/202 (94.1%)	99/107 (92.5%)	91/95 (95.8%)	*p* = 0.006
VZV-IgG	87/104 (83.7%)	48/59 (81.4%)	39/45 (86.7%)	144/152 (94.7%)	80/85 (94.1%)	64/67 (95.5%)	*p* = 0.003
CMV-IgG	66/129 (51.2%)	37/78 (47.4%)	29/51 (56.9%)	152/199 (76.4%)	79/104 (76%)	73/95 (76.8%)	*p* < 0.0001
TBC	0/126 (0%)	0/80 (0%)	0/46 (0%)	21/189 (11.1%)	15/112 (13.4%)	6/77 (7.8%)	*p* < 0.0001
