# Supplementary material for: Serology of Viral Infections and Tuberculosis Screening in an IBD Population Referred to a Tertiary Centre of Southern Italy
Source: Gastroenterol Res Pract. 2017 Sep 17;2017:4139656. doi: 10.1155/2017/4139656 (PMC5623777; doi:10.1155/2017/4139656)
Supplement: Supplementary file 1 — Supplementary Fig. 1: Seroprevalence of EBV infection by age intervals; green bars are data from our centre, grey bars reference values from Linton et al [19]. Supplementary Fig. 2: Seroprevalence of CMV infection by age intervals. [file 4139656.f1.docx]

Supplementary Fig. 1: Seroprevalence of EBV infection by age intervals; green bars are data from our centre, grey bars reference values from Linton et al [19].

Supplementary Fig. 2: Seroprevalence of CMV infection by age intervals.
